# Dr. Robert Hare

**Published:** 1858-07

**Authors:** 


### NECROLOGICAL NOTICES.

DEATH OF DR. ROBERT HARE.—The death of this eminent man took place in this city on the 16th of May, in the seventy-seventh year of his age. Even as a young man he made his name known throughout the whole world by his brilliant discoveries. He held the Chair of Chemistry in the University of Pennsylvania for nearly thirty years. As a lecturer he was not brilliant, but always conducted his experiments on a grand scale. Besides his contributions to science through the journals of the day, he wrote very little; his "Compendium of Chemistry" not being a systematic treatise, but merely a text-book for his students. During the last few years he had labored under a mental delusion concerning "Spiritualism," in which he was a firm believer. At the meridian of his life, Dr. Hare was undoubtedly the first chemist that this country has ever produced.

